# Combined Approach to Leukemic Differentiation Using Transcription Factor PU.1-Enhancing Agents

**DOI:** 10.3390/ijms23126729

**Published:** 2022-06-16

**Authors:** Petra Bašová, Helena Paszeková, Lubomír Minařík, Martina Dluhošová, Pavel Burda, Tomáš Stopka

**Affiliations:** BIOCEV, 1st Medical Faculty, Charles University, 25250 Vestec, Czech Republic; basova.petra@gmail.com (P.B.); paszekova.helena@gmail.com (H.P.); lubomir.minarik@vfn.cz (L.M.); martina.dluhosova1@gmail.com (M.D.); pavel.burda82@gmail.com (P.B.)

**Keywords:** transcription factor PU.1, microRNA miR-155, 5-Azacytidine, Celastrol

## Abstract

The transcription factor PU.1 (Purine-rich DNA binding, SPI1) is a key regulator of hematopoiesis, whose level is influenced by transcription through its enhancers and its post-transcriptional degradation via microRNA-155 (miR-155). The degree of transcriptional regulation of the *PU.1* gene is influenced by repression via DNA methylation, as well as other epigenetic factors, such as those related to progenitor maturation status, which is modulated by the transcription factor Myeloblastosis oncogene (MYB). In this work, we show that combinatorial treatment of acute myeloid leukemia (AML) cells with DNA methylation inhibitors (5-Azacytidine), MYB inhibitors (Celastrol), and anti-miR-155 (AM155) ideally leads to overproduction of PU.1. We also show that PU.1 reactivation can be compensated by miR-155 and that only a combined approach leads to sustained PU.1 derepression, even at the protein level. The triple effect on increasing PU.1 levels in myeloblasts stimulates the myeloid transcriptional program while inhibiting cell survival and proliferation, leading to partial leukemic differentiation.

## 1. Introduction

One of the hallmarks of myeloid leukemia is the progressive accumulation of clonal precursors that are prevented from developing into normal mature cells. Among the myeloid malignancies, there are chronic progressive diseases such as myeloproliferative neoplasias (MPN) and myelodysplastic syndromes (MDS), which progressively accumulate additional driver mutations and progress to AML. One strategy to inhibit leukemia is the induction of leukemic cell differentiation, a concept that involves therapeutically inducing (often by manipulating transcription factors levels) differentiation and simultaneously arresting division, which ultimately alters the aggressive behavior of the leukemia and normalizes the development of functional blood cells [1]. Normal myeloid cell development is regulated at the progenitor level by the key and indispensable transcription factor PU.1, which regulates the targets dependently to its expression level and therefore decides whether either granulocytes, monocytes, or other leukocyte subtypes will be produced [2]. In contrast to the process of leukemogenesis, during normal myelocyte development, but also in chronic inflammatory reactions or sepsis, myelopoietic regulatory factors are able to activate the transcription of the *PU.1* gene very efficiently. Previous research in mouse models has shown that a reduction in PU.1 levels represents a situation where there is a progressive block in differentiation and the development of disease that is similar to MPN, even with a propensity to develop AML [3]. In a similar but slightly different mouse model, induced conditional deletion of the *PU.1* gene led to the expansion of granulocyte–macrophage (GM) progenitor cells with impaired differentiation potential [4], which progressively develop an MPN-like disease that also includes a decrease in peripheral blood cells (cytopenia), enlargement of the spleen and liver, and hypocellular bone marrow populated predominantly by myeloblasts [4]. The regulation of PU.1 levels is indispensable for normal hematopoiesis, while reducing PU.1 below a certain threshold (20%) through mutations or downregulation promotes the development of MPN or AML in mouse models or human patients [5,6,7].

The *PU.1* gene is relatively complexly regulated at the level of several enhancers and at a magnitude of several kb prior to the start of transcription. The deletion of a particular enhancer called the URE (upstream regulatory element), which attracts a number of regulatory transcription factors, disrupts PU.1 expression, such that the URE is unable to associate with and effectively stimulate the *PU.1* promoter [8]. In addition to the transcriptional regulation of PU.1 levels, there are other mechanisms, including at the post-transcriptional level. For example, the antisense transcript of PU.1 can inhibit PU.1 expression by modulating mRNA translation [9]. Another RNA from a set of small non-coding microRNAs, called miR-155, which is expressed in hematopoietic stem and progenitor cells, can also strongly inhibit PU.1 expression, even representing an oncogenic factor mediating myeloid progenitor expansion [10] and playing an important role in leukemogenesis [11]. Another factor influencing miR-155 and PU.1 levels is the transcriptional activator MYB, which is overexpressed in immature hematopoietic cells and leukemic progenitors. Its role is to regulate proliferation and differentiation in this early cellular compartment [12]. We have demonstrated that miR-155, MYB, and PU.1 have interconnected regulatory relationships, meaning a change in the level of one of them automatically adjusts the levels of the other two factors, leading to significant phenotypic differences, as shown in two relatively distinct hematopoietic systems [13,14]. At the same time, we have previously shown that DNA methylation represents a barrier to effectively increasing PU.1 levels in a model of AML [15].

Previous studies indicated that while the deletion of PU.1 expression abolishes myeloid development, a reduction in PU.1 levels has an apparent leukemia-promoting effect [16]. The pathways by which PU.1 is reduced and the gradual activation methods of the differentiation block may be multiple, both through disruption of the transcriptional regulation of the *PU.1* gene and post-transcriptionally. Our previous work showed that miR-155 is under the influence of the oncogenic transcription factor MYB [13]. The inhibition of MYB (by Celastrol, CEL) was demonstrated to block AML growth by unlocking the interaction of MYB and p300 at the promoters of its oncogenic targets [17]. CEL is a quinone methide triterpene by chemical composition that is obtained from the extract of the thunder god vine root, and the number of its therapeutic targets is expanding. In our further work, we showed that not only by blocking miR-155 or MYB, but also by using a DNA methylation inhibitor (5-Azacytidine, AZA), we could increase PU.1 expression quite efficiently, thereby promoting myeloid cell differentiation and suppressing the leukemogenic phenotype of the cells [15]. In our current project, we built on previous hypotheses and asked whether a combined action to increase PU.1 levels can establish a leukemic differentiation effect that would be of a more sustainable character, thereby enhancing the redirection of AML cells while suppressing the leukemic phenotype.

## 2. Results

### 2.1. Myb Inhibition Has a Partial Prodifferentiation Effect Due to the Compensatory Effect of miR-155

**Figure 1:**
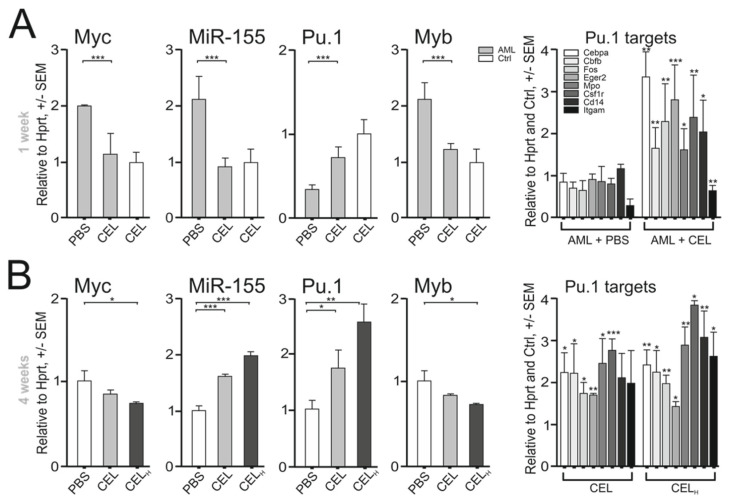

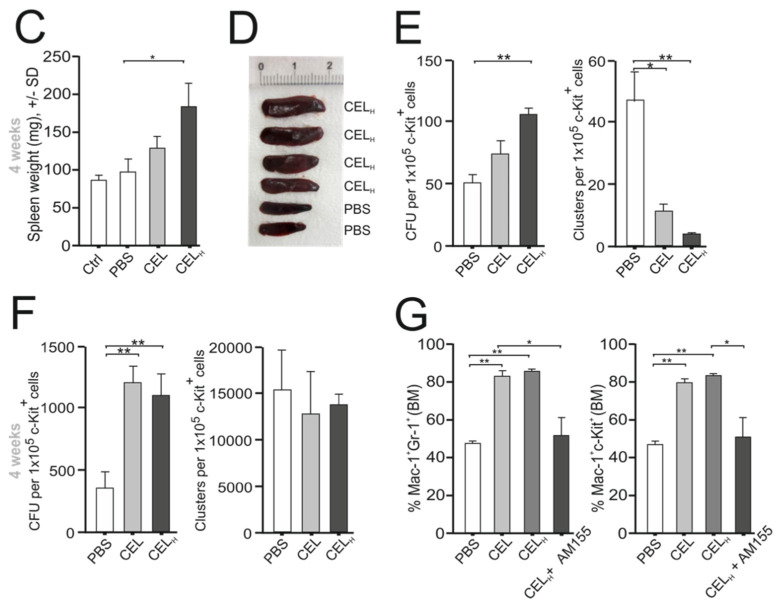
Compensatory effect of miR-155 on CEL effects on murine PU.1^ure/ure^ AML: (**A**) 1 week CEL therapy of 3 i.p. 10 μg injections, mRNA expression in BM c-Kit^+^ blasts, WT (*n* = 6), AML-bearing 5–6-month-old PU.1^(ure/ure)^ mice (*n* = 6), determined by qPCR. Fold changes (*y* axis), controls set to 1, mean ± SEM, *p*-values (*t*-test, unpaired, two-tailed); (**B**) 4 week CEL therapy with either standard weekly dose or three-fold escalated dose (H), mRNA expression of PU.1 targets normalized on AML + PBS (A); (**C**) spleen weight AVG ± SD, 4-week CEL therapy, WT (*n* = 3), PU.1^ure/ure^ (*n* = 3); (**D**) spleen photographs; (**E**) CFU and immature cell colonies (clusters) cultured from c-Kit^+^ cells of PU.1^ure/ure^ mice (*n* = 3) after 4-week CEL therapy; (**F**) CFU and immature colony cultures after replating; (**G**) effects of 4-week AM155 and CEL on progenitor cells. Flow cytometry of BM from PU.1^ure/ure^ (*n* = 3). Mean ± SEM, *p*-values (*t*-test, unpaired, two-tailed) * *p* < 0.05, ** *p* < 0.05, *** *p* < 0.005.

Based on the previous results, we first investigated whether the MYB inhibitor (CEL) affects leukemic differentiation by regulating PU.1 levels. To do this, we used a hypomorphic PU.1 model of AML/MPN in mice [3], and treated these mice upon AML development with CEL. The experimental conditions in the mice were previously optimized [17]. In order to understand the effect of the therapy at the molecular level, we monitored the consequences of CEL administration in the short (1 week) and medium (4 weeks) terms. Treatment with CEL for one week significantly reduced the levels of miR-155 as well as other CEL targets (Myb, Myc) in magnetically isolated c-Kit-positive myeloid progenitors compared to the control vehicle in treated AML-bearing 5–6-month-old PU.1^ure/ure^ or control age-matched mice (Figure 1A). As expected, the CEL treatment also affected the miR-155 and PU.1 levels in progenitor cells, similarly to what has been previously observed in vitro using Myb siRNA [13], via upregulating PU.1 targets (Figure 1A, right). With prolonged CEL treatment (for 4 weeks), the PU.1 program was further amplified (Figure 1B), but this was followed by an unexpected upregulation of miR-155 (Figure 1B). The upregulation of miR-155 represents a compensatory mechanism that activates miR-155 in progenitor cells at a certain threshold level of PU.1 [10]. The PU.1-dependent upregulation of miR-155 has previously been shown to counteract differentiation and promote a myeloproliferative state in mice [11]. Indeed, the medium-term treatment with CEL (for 4 weeks) increased the proportion of early bone marrow (BM)-derived myeloid progenitors that could not fully complete their differentiation fate, leading to their accumulation, and consequently splenic enlargement (Figure 1C,D and Supplementary Figure S1A). CEL therapy was also unable to significantly increase the numbers of mature neutrophils and monocytes in PU.1^ure/ure^ mice (Figure S1B), but we observed a trend in this direction. While a short-term treatment with CEL stimulated the growth of myeloid colonies (CFU) (Figure 1E) and blocked immature progenitor clusters (>50 cells/cluster) (Figure 1E), the medium-term therapy with CEL was unable to suppress the growth of clusters in semisolid media upon replating of progenitors (Figure 1F). This suggested that CEL therapy is compensated at the level of the progenitors, in which it is unable to fully force their differentiation. The higher proportion of early PU.1^ure/ure^ progenitors induced by medium-term CEL therapy represented the early myeloid Mac1^+^c-Kit^+^ progenitors and late monocyte–granulocytic (Mac1^+^Gr1^+^) precursors (Figure 1G). To test the role of elevated miR-155 in CEL-induced MPN-like features, we used anti-miR-155 (AM155) on top of the CEL therapy, which (as expected) significantly decreased the miR-155 level and lowered fractions of both Mac1^+^Gr1^+^ and Mac1^+^c-Kit^+^ BM-derived cells in PU.1^ure/ure^ mice (Figure 1G). In conclusion, short-term PU.1^ure/ure^-AML treatment with CEL blocked the Myb and miR-155 levels, thereby deregulating PU.1 and inducing myeloid differentiation. However, medium-term CEL therapy led to myeloid cell accumulation and splenomegaly. This phenomenon of unsustainable leukemic differentiation was attributed to the compensatory upregulation of miR-155, as suggested by the experiment in which the CEL therapy was complemented by AM155 (Figure 1G).

### 2.2. Induction of DNA Hypomethylation Leads to Increased PU.1 Level and Anti-AML Effect

**Figure 2:**
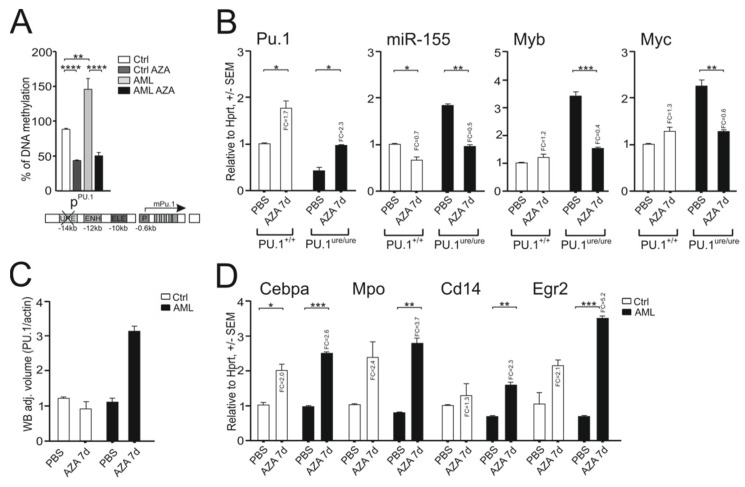

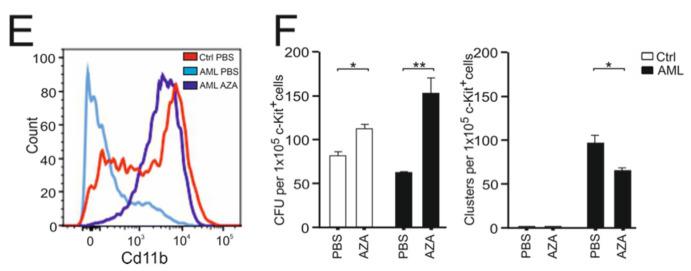
AZA derepresses the *PU.1* gene in PU.1^ure/ure^ AML: (**A**) methylation of m*PU.1* promoter following AZA for 1-week, 3 doses, sets of PU.1^(ure/ure)^ AML-bearing 5–6 month old mice and control mice were used (*n* = 4 per group); (**B**) mRNA expression by qPCR, 1 week AZA treatment in 3 doses, genotypes indicated; (**C**) Western blot (densitometry) for PU.1 protein in AZA-treated mice with indicated genotypes relative to β-actin; (**D**) mRNA expression by qPCR (same as B), 1 week AZA treatment in 3 doses, genotypes indicated; (**E**) flow cytometry of Cd11b expression in PU.1^ure/ure^ mice, either untreated (light blue) or treated with AZA (violet), or control untreated BM (red); (**F**) CFU and immature cell colonies (clusters) cultured from c-Kit^+^ cells of control or PU.1^ure/ure^ mice (*n* = 3) treated with AZA for 7 days. Mean ± SEM, *p*-values (*t*-test, unpaired, two-tailed) * *p* < 0.05, ** *p* < 0.05, *** *p* < 0.005, **** *p* < 0.0005.

In the case of the PU.1^ure/ure^ hypomorphic mutant, the level of PU.1 expression is low [3], although due to the demethylation action of AZA it can be effectively increased [15] by targeting the hypermethylated region of the PU.1 promoter in AML and also to some extent in control cells (Figure 2A). Other regions of methylation within the *PU.1* locus in AML cells and controls were relatively low. As expected, the mRNA for PU.1 was induced after AZA treatment, while the miR-155, Myb, and MYC proto-oncogene (Myc) levels were reciprocally reduced (Figure 2B). AZA was able to increase the PU.1 protein levels at the 7 day time point, as shown in the immunoblot (Figure 2C and Figure S2F). As expected, the PU.1 targets CCAAT enhancer binding protein alpha (Cebpa), myeloperoxidase (Mpo), Cd14, and early growth response 2 (Egr2) were stimulated with AZA in both control and PU.1^ure/ure^ progenitors (Figure 2D). While an effect of AZA on neutrophil maturation was observed in normal progenitors (Figure S2A), no such effect was observed in PU.1^ure/ure^ AML cells, again supporting the hypothesis that the leukemic differentiation observed at the progenitor level is unsatisfactory and results in a lack of functional specialization of neutrophil granulocytes in MDS. We used granulocyte colony stimulating factor (G-CSF) to compare the effects of AZA on myeloid progenitors. The stimulatory effects of AZA or G-CSF on PU.1 and its target mRNA levels were comparable (Figure S2B). The upregulation of Cd11b protein expression was increased after AZA similarly as with G-CSF (Figure 2E and Figure S2C). AZA (Figure 2F), G-CSF, and their combination (G-CSF added before AZA) (Figure S2D) were shown to increase the number of differentiating CFU colonies, and more importantly these treatments suppressed the growth of leukemic clusters (Figure 2F and Figure S2D), suggesting that AZA cooperates with G-CSF to induce early myeloid progenitor cell differentiation in the PU.1^ure/ure^ model. Furthermore, for two target genes, PU.1 and Egr2, we observed that the combination of AZA with G-CSF had a significant additive effect (Figure S2E). As expected, the upregulation of PU.1 by AZA also had an effect on the levels of Myb and miR-155 in a 7 day experiment (Figure 2B) in which RNA levels for Myb and miR-155 were inhibited.

**Figure 3:**
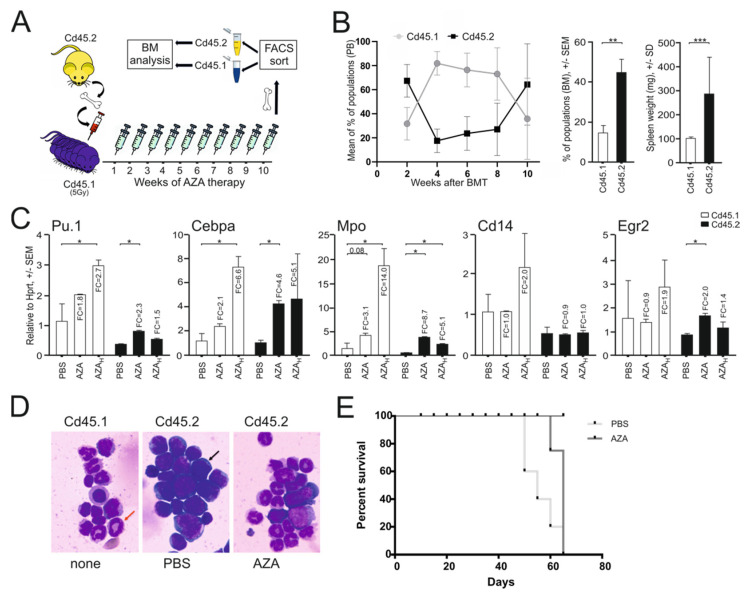
AZA therapy promotes myeloid differentiation in murine PU.1^ure/ure^ p53^−/−^ AML: (**A**) scheme of transplantation procedure and AZA therapy schedule (3x weekly for 10 weeks); (**B**) PU.1^ure/ure^ p53^(−/−)^ cells (Cd45.2) were transplanted into Cd45.1 acceptor after its sublethal irradiation (*n* = 6); far right, spleen weight AVG ± SD (*n* = 5), *p*-values (*t*-test, unpaired, two-tailed); (**C**) mRNA expression in donor (Cd45.2) and acceptor (Cd45.1) BM controls (*n* = 2) and AZA (*n* = 3) arms as determined by qPCR; *p*-values (*t*-test, unpaired, two-tailed) * *p* < 0.05, ** *p* < 0.05, *** *p* < 0.005; (**D**) cytology of spun cells stained via Giemsa-May-Grunwald protocol (dark arrow = myeloblast; red arrow: neutrophil band); (**E**) survival curves of AML (*n* = 10) and control (*n* = 4) mice; follow-up at 70 days.

To determine whether the AZA treatment inhibited leukemic growth in vivo, we transplanted highly aggressive PU.1^ure/ure^p53^−/−^ progenitor cells (expressing Cd45.2) into lethally irradiated recipient mice expressing Cd45.1. The AML engraftment of Cd45.2 donor cells lasted for the next 10 weeks and accounted for more than 60% of the BM hematopoiesis (Figure 3A,B). We also observed that xenografted mice developed marked splenomegaly (Figure 3B, right). Mice with documented PU.1^ure/ure^p53^−/−^ chimerism were then treated with AZA in two different dosing schedules that resembled that used in human MDS patients, which is also comparable to that previously tested elsewhere in mice [18]. We also evaluated the myeloid mRNA program in KIT proto-oncogene receptor tyrosine kinase (c-Kit)-positive progenitors from both donor and recipient cells. As shown in Figure 3C, AZA within 10 weeks upregulated PU.1 and its program in both normal and AML cells. The AZA treatment reduced the amount of myeloblasts visualized in cytological smears (Figure 3D), and in contrast activated the appearance of normally differentiating myeloid cells. The differentiation of AML progenitors along with the upregulation of the PU.1 program was also associated with the prolonged survival of AZA-treated transplanted mice (Figure 3E), although because of the particular experimental design, we did not measure a specific time difference in survival. Again, we confirmed that AZA is able to increase PU.1 levels in AML lacking the URE, and we also observed that this treatment additionally prolonged the survival of mice with AML. Thus, we again indirectly confirmed that PU.1 is a very important barrier to AML pathogenesis and that hypermethylation of its promoter is abrogated by AZA therapy, which may play a key role in its targeting effects.

### 2.3. Myb/miR-155 Inhibition and DNA Hypomethylation Exhibit Synergy in PU.1 Deregulation

The experiments in the previous two sections showed that by inhibiting MYB, miR-155, and DNA methylation, the PU.1 levels become upregulated in the PU.1^ure/ure^ mouse model of AML through different mechanisms. We also observed that the upregulation of PU.1 was able to stimulate the myeloid transcriptional program but was unable to reach its maximal level and force progenitor cells to differentiate sustainably or cause the mature cells. We asked whether by combining different mechanisms we could achieve a maximal effect on PU.1 deregulation. We took advantage of the fact that human AML cell lines with previously documented effects of MYB and miR-155 inhibitors led to the upregulation of PU.1 levels [13], and tested the combination of the 3 drugs using these cellular systems.

**Figure 4:**
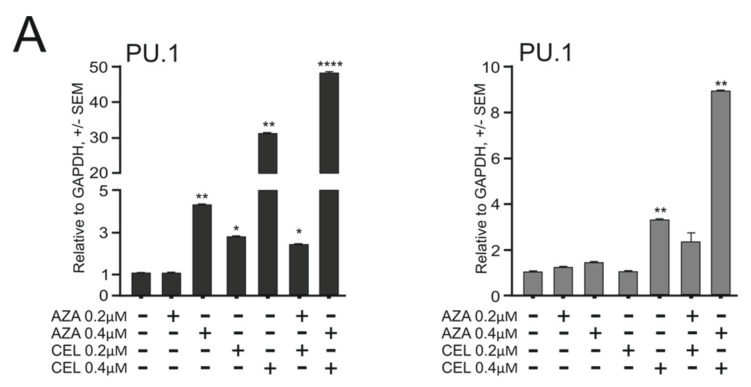

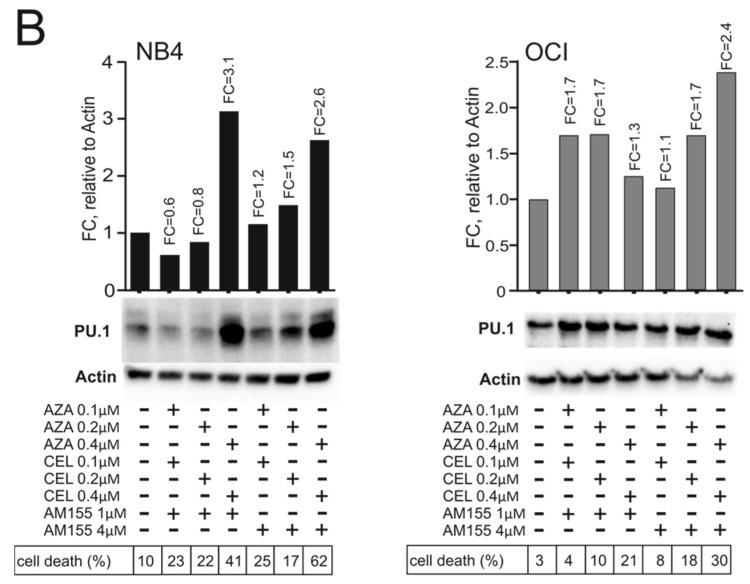

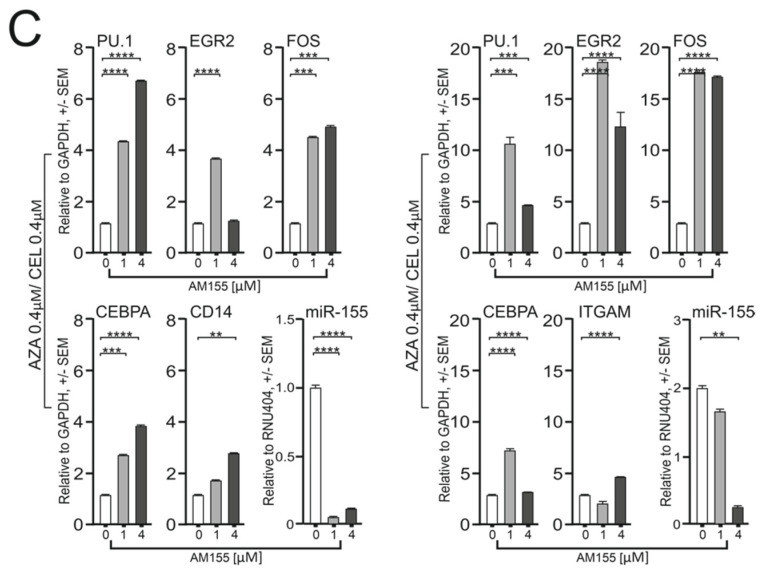
Combined CEL/AZA/AM155 therapy induces PU.1 program in human AML. Triplicate experiments, NB4 cells shown on left, OCI-M2 on right: (**A**) mRNA expression of PU.1, 72 h treatment of indicated agents and their combinations, final concentrations indicated; (**B**) protein levels of PU.1 and β-actin upon 72 h therapy with AZA, CEL, AM155; μM concentrations indicated, cell death % shown by the table; (**C**) mRNA expression at 72 h upon treatment with 0.4 μM AZA and 0.4 μM CEL. On top are 1 μM and 4 μM AM155. Mean ± SEM, significance indicated by star (* *p* < 0.05, ** *p* < 0.05, *** *p* < 0.005, **** *p* < 0.0005).

**Figure 5:**
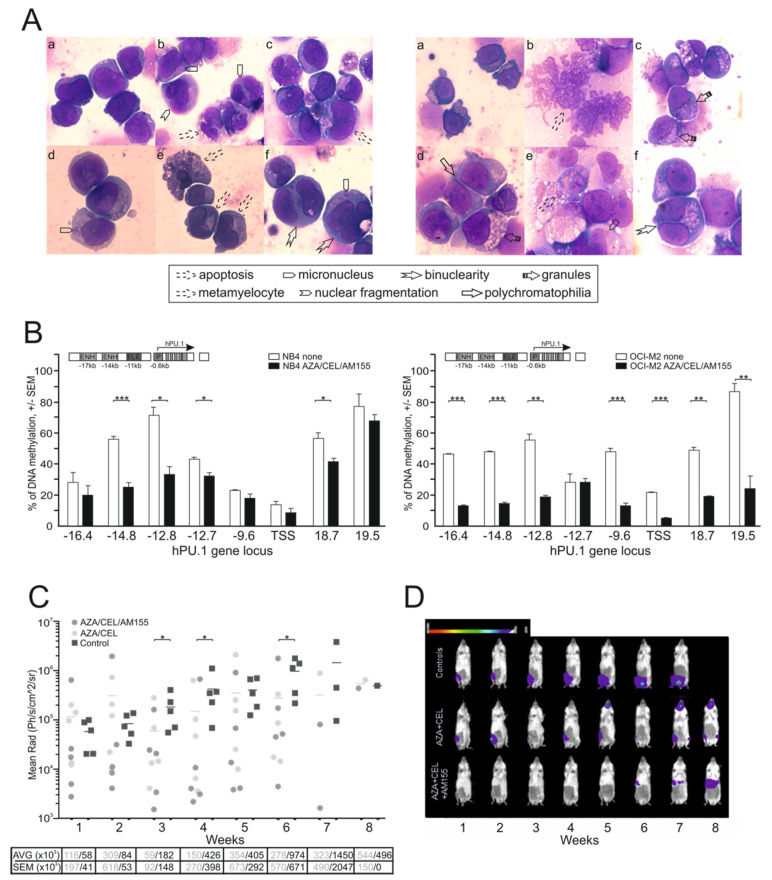
Evaluating the effects of combined therapy with CEL, AZA, and AM155. (**A**) Cytology staining via standard May–Grünwald–Giemsa-Romanowski protocol after exposing the hAML cell line NB4 (left) and OCI-M2 cell line (right) for 72 h to 0.4 μM AZA, 0.4 μM CEL, and 4 μM AM155 (**b**–**f**) compared to uniform myeloblasts or proerythroblasts respectively seen in untreated (**a**) controls. (**B**) DNA methylation of hPU.1 genes, untreated (empty) or treated for 72 h with 0.4 μM AZA, 0.4 μM CEL, and 4 μM AM155 (dark). Methyl DNA IP assay (MagMeDIP), *y*-axis = %methylation; *x*-axis = DNA loci. Unpaired two-tailed Student’s *t*-test was used. Error bars represent standard deviations. (**C**) Analysis of luciferase activity in mice treated with either 3-drug combination or a vehicle; see Section 2.3. Mean is shown, unpaired Mann–Whitney *t*-test. Table below provides AVG and SEM values; *p*-values: * *p* < 0.05, ** *p* < 0.05, *** *p* < 0.005. (**D**) Examples from the 3 experimental groups (*y*-axis); luminescence weekly under general anesthesia (isoflurane) after i.p. addition of D-luciferin, see Materials and Methods.

First, we defined the inhibitory concentration (IC) with 50% effect (using the WST1 assay) for CEL and AZA in two human AML cell lines (NB4, OCI-M2). In the case of AM155, we observed that there was no cytotoxic effect at any level. We used the WST1 assay to test the effects of the triple combination of the selected agents. First, we combined CEL and AZA at different concentrations and observed that the two modalities synergistically elevated PU.1 mRNA (Figure 4A and Figure S3B). Next, we used doses of CEL and AZA that had a synergistic effect and added an additional factor, AM155 (at 1 and 4 μM concentrations). We observed that the addition of AM155 was capable of further increasing the PU.1 protein level (Figure 4B and Figure S3C,D). However, despite the stimulatory effect at the transcriptional level, the combination of AZA + CEL was unable to markedly increase PU.1 protein, again implicating a role of miR-155 in post-translationally blocking PU.1, and only after adding the AM155 did PU.1 protein production occur at an increased level. The effect of AM155 on miR-155 levels is shown in Figure 4C. We observed that all three agents independently, but especially AM155, led to an increase in the proportion of cells in the G1 phase in myeloblasts, with a concomitant decrease in proliferating cells in the S phase (Figure S3E). Coincidentally, the proportion of cell death was increased in the 3-drug combination, indicating that AML cells are unable to fully tolerate forced differentiation and induced apoptosis at elevated PU.1 levels, which fulfils the paradigm of a state of forced leukemic differentiation (Figure S3A,F). Our findings are further supported by our cytological examination of the treated cells (Figure 5A), which confirmed the induction of differentiation (polychromatophilia, higher number of metamyelocytes from myeloblasts, cytoplasmic granules), pre-apoptosis (with nuclear fragmentation, cytoplasmic vacuoles), and partial dysplasia with nuclear micronuclei and binucleation. We further evaluated the DNA methylation at the PU.1 regulatory locus, specifically at the URE, as well as in other enhancers and promoters in both human AML cell lines (NB4 and OCI-M2). Both of these lines showed the hypermethylation in the enhancer region of the *PU.1* gene prior to the treatment, with OCI-M2 having a wider region of hypermethylation (−16.4 kb to +19.5 kb) and NB4 having a relatively smaller hypermethylated region (−14.8 kb to +18.7 kb) (Figure 5B). The 3-drug combination effect on DNA demethylation, mainly imposed by AZA, was observed in several regions, including URE −16.4, −14.8, and −12.8, −12.7, but also in the 3′ region (possibly as a result of the distal effect of AZA) (Figure 5B). Thus, the derepression of the PU.1 protein alongside the leukemic growth arrest occurs upon the combined modulation of the *PU.1* gene transcription, as well as post-transcriptionally.

To investigate whether combined treatment with AZA, CEL, and AM155 can inhibit tumor growth in vivo, we used an in vivo methodology to monitor tumor growth in cell-line-derived xenograft (CDX) mice enabled via the intravital detection of a luciferase signal ectopically expressed in tumor cells engrafted within the immunodeficient (non-obese diabetic/severe combined immunodeficiency) NOD/SCID gamma (NSGS) mice. As expected, we observed the engraftment of the tumor tissue from transplanted OCI-M2 cells and verified that tumors derived from CDX mice exhibit the typical cytology of the OCI-M2 cells used for transplantation, i.e., they exhibit typical features of myeloblasts with fine chromatin and bulky nuclei, and we also confirmed via flow cytometry the concordance of their surface antigens (including the leukocyte-specific feature, CD45). Tumors visualized via luciferase detection from tumor cells showed intraosseous localization and typically linearly expanding growth. Furthermore, we applied the therapy regimen of AZA (100 μg/mouse) and CEL (100 μg/mouse) +/− AM155 (LNA-modified ATTACGAT, 225 μg/mouse) 3 times a week i.p. compared to vehicle-treated mice. Luciferase detection was performed once a week. From the data involving two groups of mice (treated with the therapeutic combination vs. the control vehicle, PBS), we observed statistically significant reductions in tumor mass (Figure 5C,D and Figure S4A,B). The luciferase detection monitoring revealed that the provided therapies can specifically and effectively inhibit xenografted tumor tissue outgrowth.

## 3. Discussion

Our previous work showed that the deregulation of miR-155 decreased PU.1 levels and that the activation of MYB led to increased aggressiveness of AML [13]. Therefore, we based our strategy on reversing this condition. We affected the PU.1 protein levels by exploiting the antileukemic effect of CEL, which is known to inhibit the transcription of MYB-dependent target genes, including mRNA for Myc [17]. CEL also prolongs the survival of mice in aggressive AML models based on the retrovirally induced expression of the fusion protein MLL-AF9 or c-Myc/Bcl2 in hematopoietic progenitor cells [17]. We have shown that the transcription factor PU.1 is also among the gene targets of CEL action, and while CEL temporarily increases its expression (including upregulation of its target program), our experiments also showed that increased PU.1 levels are compensated by increased miR-155 levels. As a result, the differentiating cells proliferate and accumulate in the spleen. While a number of studies have demonstrated a very effective role of CEL in terms of its antileukemic effect [19], we have noted here how tumor cells compensate for this inhibitory effect. It should also be noted that CEL has a relatively complex effect, as it acts not only on Myb but also on C/EBPbeta by interfering with the interactions of both transcription factors with p300. Furthermore, Myb, C/EBPbeta, and p300 are known to cooperate in the expression of myeloid-specific genes, and the antileukemic effect of CEL also involves a myeloid-specific effect [20]. However, this antileukemic effect must include a reduction in the proliferation and survival of leukemic cells. The oncogenic activities of miR-155 are known to include stimulation of the hematopoietic stem and progenitor cell proliferation and promotion of myeloid cell expansion induced by FLT3-ITD through targeting C/EBPbeta [21].

The compensatory role of miR-155, which likely occurs when the progenitor cell takes the path of differentiation, is supported by work suggesting that the upregulation of PU.1 can directly induce the transcription of the host gene for miR-155 (*MiR155hg*). Additionally, PU.1 directly controls the expression of three other miRs (miR-146a, miR-342, miR-338) through the dynamic occupancy of binding sites in regulatory chromatin regions adjacent to their genomic coding loci [10]. Thus, if these different evidence pathways can be linked, and if this can be observed in the expression dynamics, any alterations in the Myb, miR-155, and PU.1 axis must lead to a realignment of gene expression of individual members of this pathway. However, it must also be perceived that while PU.1 activates the *MiR155hg* promoter, the mature miR-155 reduces PU.1 protein levels. Conversely, the upregulation of Myb leads to a silencing of PU.1 expression, and similarly PU.1 is one of the repressors of Myb mRNA transcription. Thus, in order to be able to block this nicely matching system, it is important to pull several ends at the same time. Specifically, one must block miR-155 or Myb production and at the same time unblock the repressive action of chromatin in the *PU.1* gene region. Therefore, the effects of AZA were utilized in *PU.1* derepression, complementing the inhibition of MYB and at the same time inhibiting miR-155.

One of the key questions regarding *PU.1* derepression is whether it results in increased PU.1 protein levels and whether the effect at the level of its molecular targets is also linked to inhibition of cell division and cell survival. Our work shows that while AZA and CEL have an effect in terms of their molecular phenotype, where indeed PU.1 derepression occurs at the mRNA level along with deregulation of the PU.1 mRNA program, the actual strong derepression of PU.1 protein levels occurs only after cells are treated simultaneously with AZA, CEL, and AM155. Although the effect of the 3-drug combination in increasing PU.1 levels as much as possible remains probably the most important step in this strategy, it cannot be excluded that the addition of AM155 (to CEL and AZA) has synergistic cell cycle blocking and proapoptotic effects through other miR-155 targets besides PU.1. However, it is known that ectopic and strong overproduction of PU.1 in AML blasts can be associated with both cell cycle arrest and apoptosis, as shown after retroviral transduction with a vector carrying PU.1, which together document the so-called restored partial myeloblast differentiation [22]. On the other hand, the ectopic and uninhibited expression of PU.1 also has the effect of inhibiting differentiation into the erythroid lineage [23], so it is possible that the activation of the myeloid program in AML may have a partially erythro-suppressive effect. To summarize, only after strong overproduction of PU.1 does leukemic differentiation occur, whereas weaker overproduction has essentially a partial effect as a sort of “molecular phenotype” with no apparent antileukemic effect. Thus, the addition of AM155 is extremely important, as it appears to decrease the fraction of myeloblasts in the S phase while the population increases in the G1 phase. In contrast, AZA monotherapy leads to an increase in the population of cells with early signs of apoptosis. Thus, with the combination of AM155, AZA, and CEL, it is not very surprising that in addition to the activation of the PU.1 transcriptional program, cells in the G1 phase have the opportunity to differentiate under the influence of the myeloid program; however, this results in the induction of cell death with any minor disturbance in the sequence of differentiation steps, probably under the influence of mutations in other key genes. Therefore, we believe that only by combining different agents with the effect of increasing PU.1 levels can we effectively inhibit the growth of myeloid malignant cells (e.g., in AML).

The question remains of how and whether the strategy of leukemic differentiation will be applied in the clinic, and furthermore whether the concept used in our paper can be applied in whole or in part to the relatively broad and heterogeneous field of MDS and AML. There already exist strategies in use today that fit the principles we have presented. An example is a peptidomimetic inhibitor that blocks the formation of the complex between MYB and CREB-binding protein (CBP/p300), which ultimately leads to the inhibition of the MYB targets—MYC and B cell leukemia/lymphoma 2 (BCL2) [24]. Since MYB is indeed a very tempting target for the development of new therapeutics, new small inhibitors are currently being developed, as exemplified by the identification of teniposide and etoposide, two chemotherapeutics used in the clinic, as apparent inhibitors of MYB [25]. The newly discovered MYB inhibitors also include monensin, which has obvious antileukemic effects [26]. Nanocomplexes containing AM155 have even been created and used for the in vitro therapy of hepatocellular carcinoma, in which they suppressed the tumor cell proliferation, migration, and induction of apoptosis [27]. The use of AM155, so far for experimental purposes, has been mostly validated for chronic lymphocytic leukemia cells in which the suppression of miR-155 (or miR-26A or miR-130a) leads to the induction of apoptosis [28]. It seems that the key will not only be to specifically target the sequence of AM155 in question, but also the method of delivery of AM155 to the target tissue will need to be addressed. Nanoparticles in combination with a nuclear localization signal seem to be very useful for this [29]. We believe that by adding agents that block the negative regulation of PU.1 expression, i.e., MYB and miR-155 inhibitors, to AZA, we can achieve leukemic differentiation, as we have demonstrated in both cellular and murine CDX models. At the same time, we show that insufficient PU.1 stimulation can lead to myeloproliferation and splenomegaly. We propose a previously unexplored strategy for the treatment of myeloid malignancies that combines different agents to both limit the compensatory mechanisms that limit PU.1 levels while transcriptionally stimulating PU.1 expression, which together induce leukemic differentiation.

## 4. Materials and Methods

Cell lines: The OCI-M2 cell line was originally derived from a 56-year-old MDS-EB2 patient in transition to AML (DSMZ collection, Braunschweig, D, EU, #ACC 619, *please note that OCI-M2 is a completely different line from the similarly named OCI-AML2*). We also used the NB4 cell line that was derived from a patient with acute promyelocytic leukemia and who displayed the characteristic translocation (15:17) (obtained from ATCC, Manassas, VA, USA). The WST-1 method for assessing cell proliferation (Roche, Basel, Switzerland) was used to obtain IC50. Cells were also stained with trypan blue solution. The number of total, live, and dead cells was measured using a Luna IITM Automated Cell Counter (Logos Biosystems, Annandale, VA, USA).

Mice: We used the PU.^1ure/ure^ hypomorph mutant, in which PU.1 expression levels were held at ~20% by the removal of the URE enhancer, which is required for promoter and URE self-stimulation [3]. The handling of the mice was concordant with our previous experimentation [13]. As a host for xenotransplantation, we used the NSGS mouse clone with genetically modified production of IL3, SCF, and GM-CSF to support xenotransplantation outgrowth. The transplantation was intraosseous into the femoral bone [30], with OCI-M2 cells bearing luciferase expression to easily monitor tumor cell expansion once a week under general anesthesia with isoflurane. D-luciferin at a volume of 100 μL was injected i.p. (intraperitoneally) prior to luminescence detection in the warming plate in a SPECTRAL Lago X Imaging System. Data for the calculation of the radiance were obtained from Spectral Instruments Imaging by Aura Imaging Software. The radiance is a calibrated absolute measurement of the photon emission from the subject (photons/second/cm²/steradian). The mean rad is defined as the total radiance/number of pixels in the ROI (region of interest), defined as the image area quantified. The Institutional Ethical Committee approved this project under #63-2020-UMG-P_Kralova_Viziova, entitled The Study of Targeted Therapy In Vivo in a PDX Model of Chemo-Resistant Myeloid Malignancies. Xenografts were monitored via imaging, as established elsewhere [31], while therapy was performed in concordance with ethical guidelines. For BM reconstitution experiments, 10^7^ BM cells from PU.^1ure/ure^ p53^(−/−)^ Ly5.2 (CD45.2) mice were transplanted into lethally irradiated (7.5 Gy) adult (8 weeks) C57BL/6J Ly5.1 control recipients. At the post-transplantation stage, the mice were tested for the presence of donor-derived cells using a flow cytometric analysis for CD45.1 and CD45.2 antigens. Upon treatment, the BM cells were isolated and sorted for c-Kit and subsequently lysed for mRNA analyses.

Methyl DNA IP assay (MagMeDIP, Diagenode, Liege, B, EU Cat. No. C02010020): The DNA was isolated using a DNeasy Blood and Tissue Kit (Qiagen, Hilden, Germany). The amplicons evaluated via qPCR covered highly conserved and DNA-methylation-sensitive loci from distal enhancers (−16 kb upstream TSS) up to the +19.5 kb locus within the *PU.1* gene. The data represent the % of the fully methylated DNA at the testis-specific H2B histone gene (TSH2B, equalized to 100%) relative to the input DNA. All data sets were compared using one-way ANOVA, Kruskal–Wallis two-tailed tests, a *t*-test (unpaired, two-tailed), and Spearman’s correlation (confidence intervals 95%).

Expression: The RNA was isolated using the QIAzol^®^ Lysis reagent (Qiagen, Hilden, Germany) and evaluated on a Nano-Drop ND1000 Spectrophotometer (Thermo Fisher Scientific, Waltham, MA, USA). A High-Capacity cDNA Reverse Transcription Kit supplemented with miR-specific primers (Thermo Fisher Scientific, Waltham, MA, USA) produced a cDNA template for PCR on LightCycler Version 1.5.0.SP3 software with a 480 real-time PCR system (Roche, Basel, Switzerland) in 384-well plates. A run consisted of 40 cycles of 95 °C for 15 s and 60 °C for 1 min. For mRNA analyses, the TaqMan probe library (Roche, Basel, Switzerland) was used. For immunoblotting, BM cells were lysed in a RIPA buffer with protease and phosphatase inhibitors (Roche, Basel, Switzerland). Denatured cell lysates were run on gradient 8–16% Mini-PROTEAN TGX Stain-Free protein gels in a Mini-Protean Electrophoresis system and dry-blotted onto a PVDF membrane using a Trans-Blot Turbo transfer system (all Bio-Rad, Hercules, CA, USA). The PVDF membrane was blocked for 1 h in 5% non-fat milk in 1 × xTBS/0,1% Tween-20 (TBST) and incubated with primary antibodies: PU.1 (Abcam, Cambridge, UK, #ab88082) and β-actin (Santa Cruz Biotechnology, Dallas, TX, USA #sc-1616-R) O/N at 4 °C. Horseradish-peroxidase-conjugated secondary antibodies (anti-mouse, anti-goat) were used to visualize bands using Westar Supernova ECL substrate (Cyanagen, Bolgna, Italy).

## 5. Conclusions

We have shown that the inhibition of the transcription factor MYB can increase PU.1 levels and induce its transcriptional program in a mouse model of AML (PU.1^ure/ure^). However, prolonged CEL treatment is unable to maintain this effect, and the compensatory upregulation of miR-155 results in accentuated myeloproliferation and splenomegaly. Thus, blocking miR-155 with AM155 represents a way to maintain the enhanced and pro-differentiation role of PU.1 in myeloid progenitors in the long term. Furthermore, we showed that AZA was also able to increase PU.1 levels at the same time that this treatment additionally prolonged the survival of mice with AML. Thus, we also indirectly confirmed that PU.1 is a very important obstacle in the pathogenesis of AML and that the hypermethylation of its promoter may play a role in the AML phenotype. By combining the aforementioned approaches, i.e., using AZA, CEL, and AM155, we were able to effectively inhibit AML growth in vitro and in vivo in CDX mice. Thus, we believe that the derepression of PU.1 protein levels and the inhibition of cell growth and survival may be modulated via the combined modification of the transcriptional level of the PU.1 gene and post-transcriptionally via inhibiting the miR-155 level.

## Data Availability

Raw data are available on request, no OMICS data has been utilized.

## Supplementary Materials

The following are available online at https://www.mdpi.com/article/10.3390/ijms23126729/s1.
