# Supplementary material for: Combined Approach to Leukemic Differentiation Using Transcription Factor PU.1-Enhancing Agents
Source: Int J Mol Sci. 2022 Jun 16;23(12):6729. doi: 10.3390/ijms23126729 (PMC9224232; doi:10.3390/ijms23126729)
Supplement: Supplementary file 1 [file ijms-23-06729-s001.zip › ijms-1542760-supplementary.pdf]

Supplementary information for:

Combined approach to leukemic differentiation using transcription factor PU.1 enhancing agents.

Petra Bašová [1], Helena Paszeková [1], Lubomír Minařík [1], Martina Dluhošová [1], Pavel Burda [1], and Tomáš Stopka [1*]

Supplementary materials and methods:

WST1 method:

For the spectrophotometric quantification of cell proliferation and viability of NB4 and OCI-M2 cells, 10uL of Cell Proliferation Reagent WST-1 (Roche, Switzerland) was used in 100uL/well in the 96-well-plate format. Measurement of the absorbance after 1 hour was performed by the ELISA reader (Tecan).

Haematopoietic Colony Assays (or CFU Blasts):

c-Kit^{+} cells from mutant mice PU.1$^{ure/ure}$ were washed in Iscove´s MDM plus 2% FBS and cultured for 12 days (10^{4}cells/1ml Methocult M3434/well) in methylcellulose medium supplied by StemCell Technologies, Vancouver, B.C., Canada. CFU and colonies of immature cells were scored independently by two researchers.

Mice and cell separation:

Anaesthesia was done with diethylether. Peripheral blood counts were determined using Advia™60. Bone marrow (BM) was flushed from femurs with 1% of BSA-PBS. Erythrocytes were lyzed with 0.15M NH_4Cl, 17mM NaCl, 0.1mM EDTA. AutoMACS (Miltenyi Biotec, Cologne, Germany) procedure: primary FITC-labeled anti-c-Kit (BioLegend, San Diego, CA, USA, clone 2B8, cat. No. 105806) and secondary anti-FITC MicroBeads (MACS, Miltenyi Biotec, Cologne, Germany, cat. no. 130-048-701).

FACS analysis:

Canto II analyzer or BD LSRFortessa™ Cell Analyzer and FACSDiva software v.6.1 (Becton Dickinson, USA). FACS antibodies (BioLegend, San Diego, CA, USA): anti-mouse monoclonal Abs anti Mac-1/CD11b (APC) (clone M1/70, cat. No. 101212), anti Gr-1 (FITC) (clone RB6-8C5, cat. No. 108406), anti c-

Kit/CD117 (PE) (clone 2B8, cat. No. 105808), Annexin V (FITC) (cat. No. 640906), EdU (Click-iT™ Plus EdU Alexa Fluor™ 488 Flow Cytometry Assay Kit, Invitrogen).

Supplementary Figures:

Supplementary Figure S1A

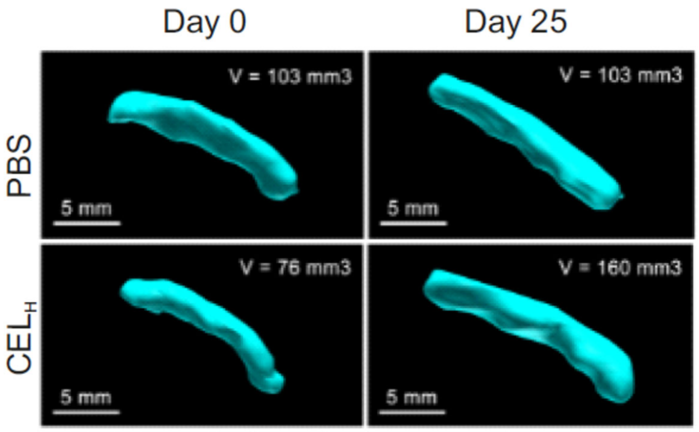

SFS1A legend: Bioimaging of the spleen in mice PU.1$^{ure/ure}$ following the 3-week CEL therapy. A representative measurement is shown from the experiment involving therapy using either CEL (N=3) or vehicle (PBS, N=3)) via i.p. injections. CEL (Celastrol, Sigma-Aldrich) (Uttarkar, Dasse et al. 2016) was administered 3 times a week via intraperitoneal (i.p.) injection in volume of 200 µl PBS in doses 30µg per injection for 3 weeks. As indicated in the methods, we induced anaesthesia in mice with isoflurane (Aerrane, Baxter, UK) - 3% for anaesthesia and 2% for maintenance of anaesthesia. Mice were depilated on the left flank (Veet depilatory cream, Reckitt, UK) and washed thoroughly with warm water. Mice were placed right side up on a heated pad and the spleens scanned along its entire length in transverse sections 50 micrometers apart (with automatic displacement). Spleen volume measurements were taken with a Vevo 3100 instrument (FUJIFILM VisualSonics, Inc.) and MX400 probe and then analyzed using Vevo LAB V.3.2.5 software (FUJIFILM VisualSonics, Inc.).

Supplementary Figure S1B

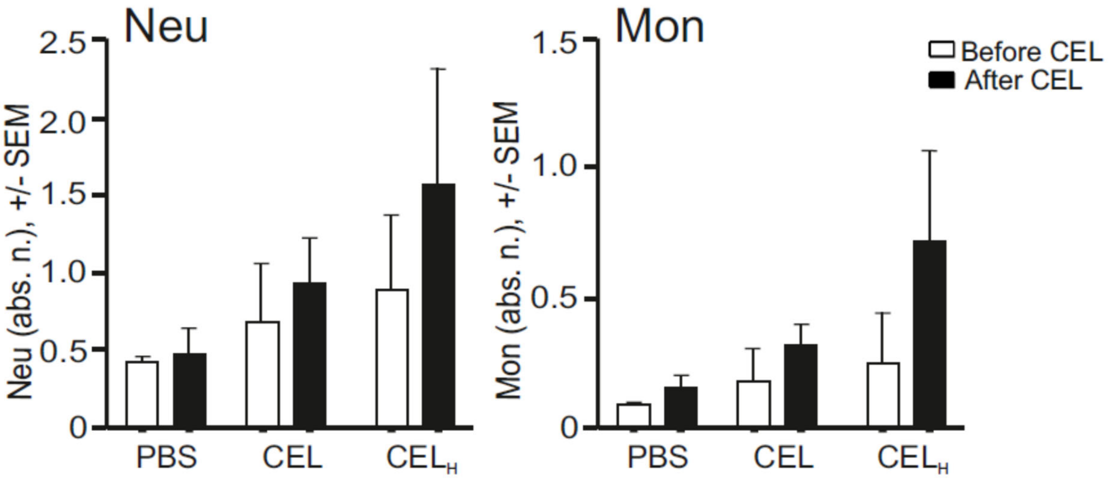

SFS1B legend: Evaluation at 4 weeks of mature neutrophils and monocytes in $PU.1^{ure/ure}$ mice either treated with vehicle or with CEL at doses 10 or 30µg adminstred via i.p. injections 3 times a week (PBS (N=3), CEL (N=3), CEL_H (N=3)). Mean ± SEM, *P*-values (t-test, unpaired, two-tailed).

Supplementary Figure S1C

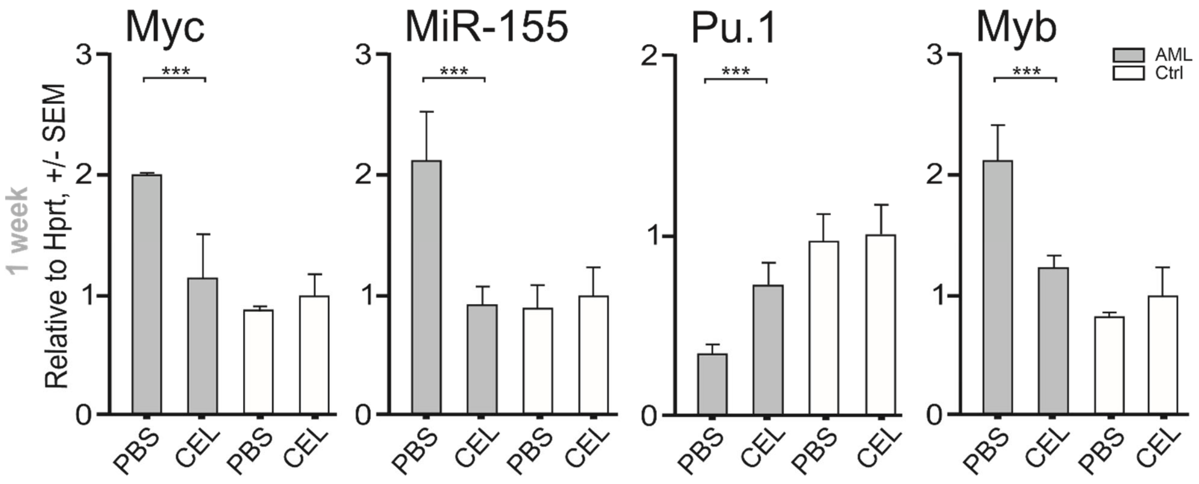

SFS1C legend: 1-week CEL therapy of 3 i.p. 10µg injections, mRNA expression in BM $c\text{-}Kit^+$ blasts, WT (N=6), AML-bearing 5-6 month old $PU.1^{(ure/ure)}$ (N=6), determined by qPCR. Fold change (y axis), controls set to 1, Mean ± SEM, p-values (t-test, unpaired, two-tailed). SHorter version of this figure presented as Fig 1A.

Supplementary Figure S2A

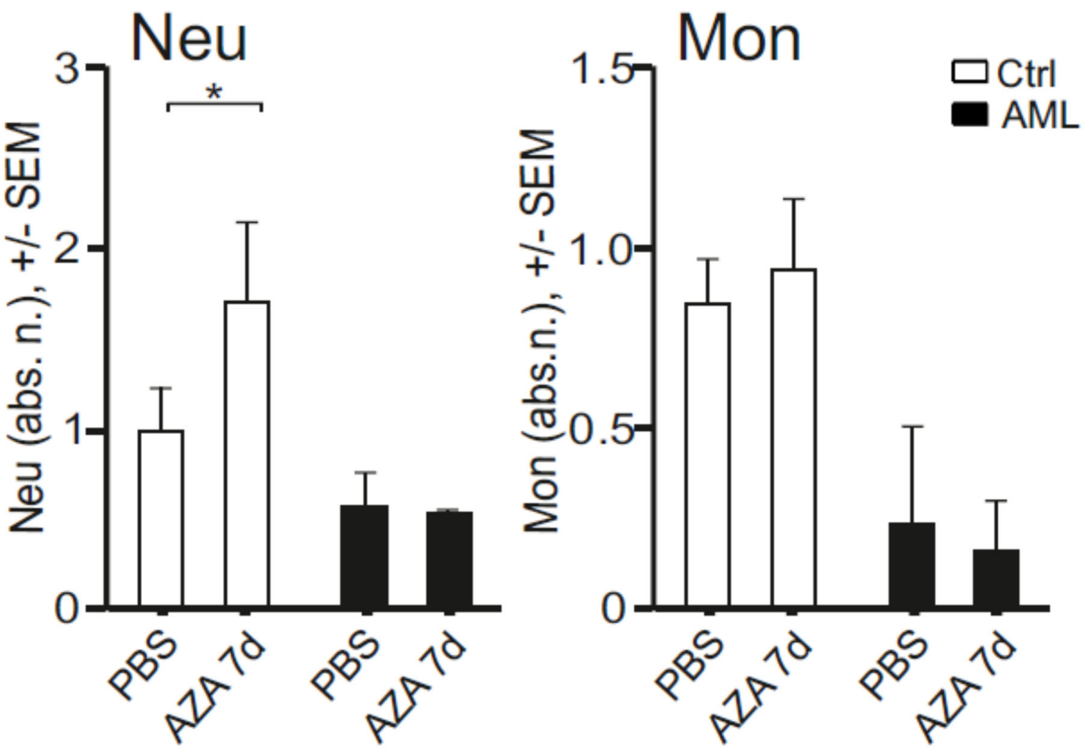

SFS2A legend: Evaluation of mature neutrophils and monocytes in PU.1$^{ure/ure}$ and ctrl mice either inejcted only with vehicle PBS (N=3) or with AZA treatment (N=3) at a dose 150 µg/mouse via i.p. once a week. Ctrl (*N*=6), PU.1$^{ure/ure}$ (N=6). Mean ± SEM, *P*-values (t-test, unpaired, two-tailed). AZA (Vidaza, Celgene) (Bejar, Lord et al. 2014) was administered once a week via i.p. injection of 150 µg/mouse in a volume of 200 µl PBS.

Supplementary Figure S2B

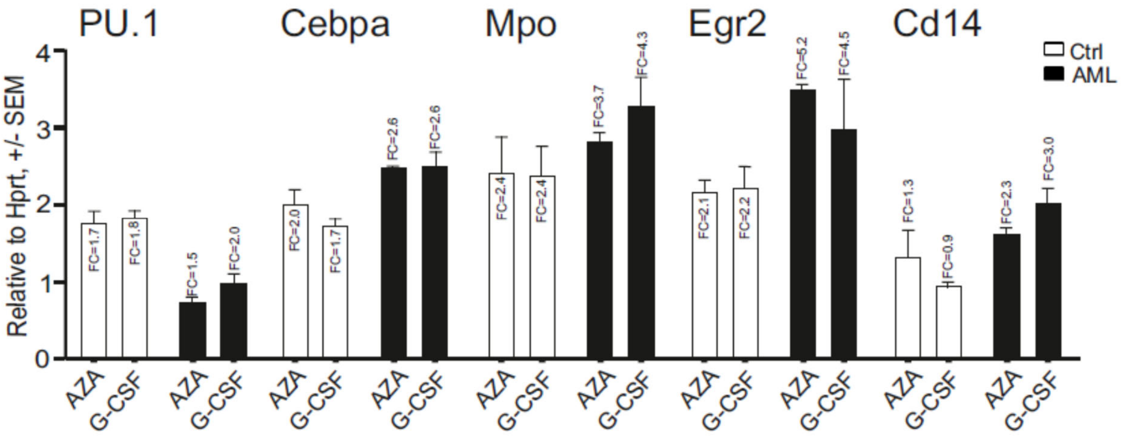

SFS2B legend: Effects of AZA or G-CSF on mRNA levels of PU.1 and its target mRNAs indicated on top. mRNA expression by qPCR. Emty bars are wild type mice and dark bars represent PU.$1^{ure/ure}$ mice. c-kit+ pozitive cells were isolated after a 7-day experiment for mRNA analysis. Therapy groups are indicated on X axis: AZA treatment (N=3) was in dose 150 µg/mouse via i.p., GCSF treatment (N=3) in dose of 5 µg/mouse via s.c. for 7 days. Ctrl (*N*=15), PU.$1^{ure/ure}$ (N=15), Mean ± SEM, *P*-values (*t*-test, unpaired, two-tailed).

Supplementary Figure S2C

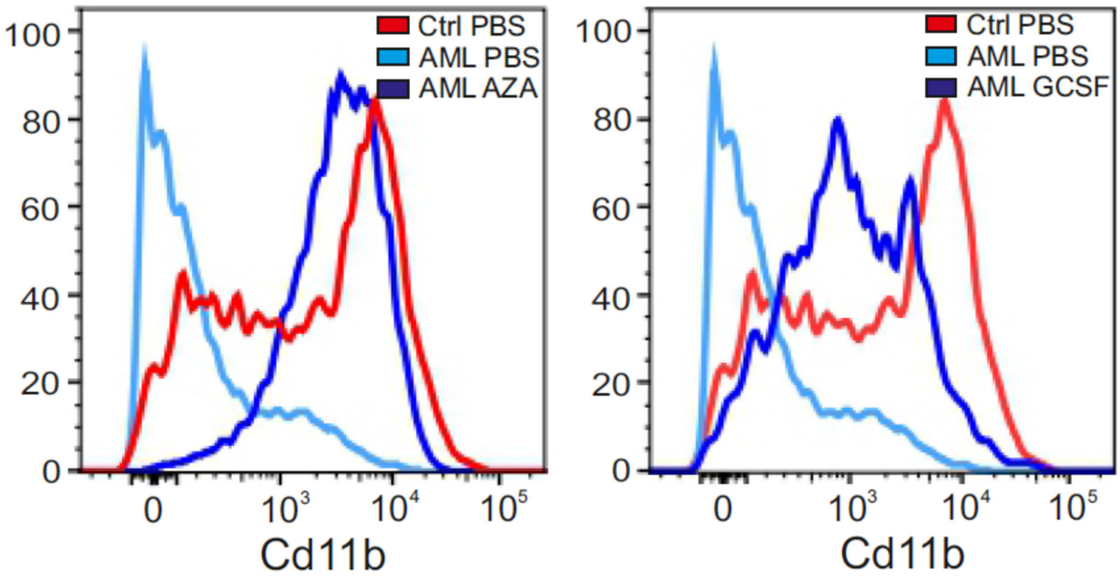

SFS2C legend: Flow cytometry analysis of CD11b expression in the PU.1$^{ure/ure}$ mice either untreated BM (light blue), control untreated BM (red), or (left) treated with AZA in dose 50 µg/mouse (violet) or (right) treated with G-CSF in dose of 5 µg/mouse (violet) for 7 days. AML indicates the PU.1$^{ure/ure}$ mice upon development of AML. Ctrl are wt mouse.

Supplementary Figure S2D

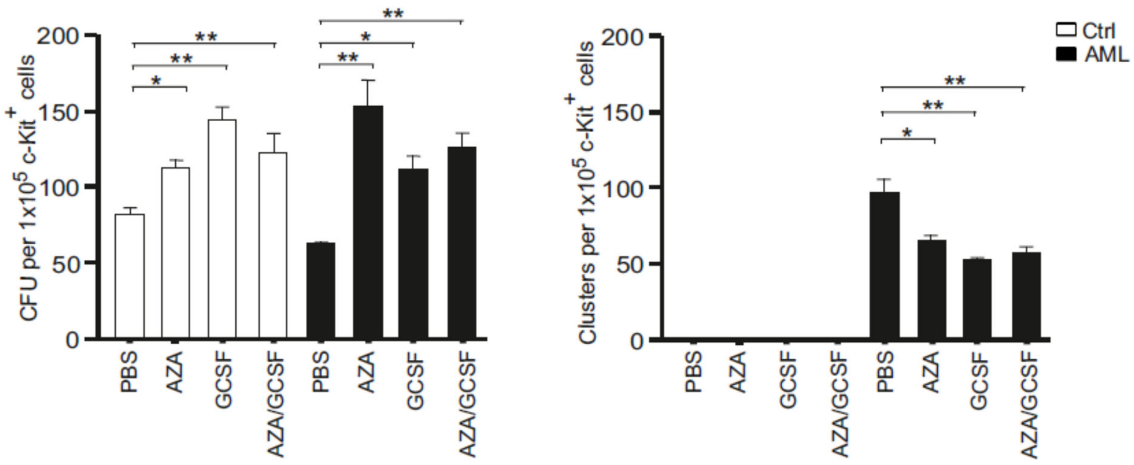

SFS2D legend: AZA and G-CSF treatment of murine AML. CFU and immature cell colonies (clusters) cultured from c-Kit$^+$ cells of control or PU.1$^{ure/ure}$ mice treated with PBS, AZA (150 µg/mouse) and G-CSF (5 µg/mouse) or their combinations for 7 days. Colonies were counted after 8 days of culture in semisolid methylcellulose medium. G-CSF in case of combination with AZA was administered 5 h before AZA. The control arm with PBS was identical to the GA arm. See M&M for more details. Mean ± SEM, *P*-values (*t*-test, unpaired, two-tailed). AML indicates the PU.1$^{ure/ure}$ mice upon development of AML. Ctrl indicates normal wt mouse.

Supplementary Figure S2E

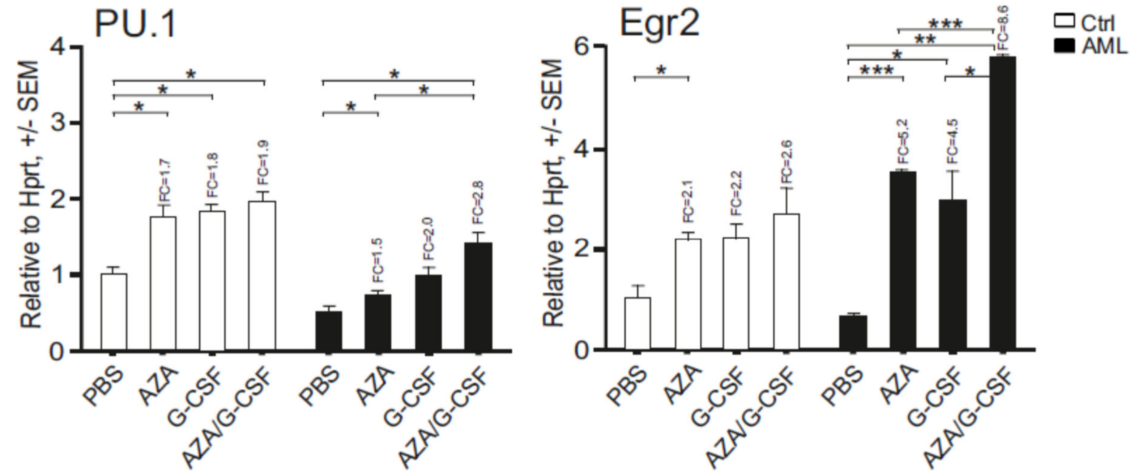

SFS2E legend: mRNA analysis of the experiment shown in SF2B-D. AZA (150 µg/mouse) and G-CSF (5 µg/mouse) treatment of murine AML. PU.1$^{ure/ure}$ mice (N=3) either treated with vehicle or with AZA (N=3) and G-CSF (N=3) or their combinations (N=3)

for 7 days. For detail see M&M section. Mean ± SEM, P-values (t-test, unpaired, two-tailed).

Supplementary Figure S2F

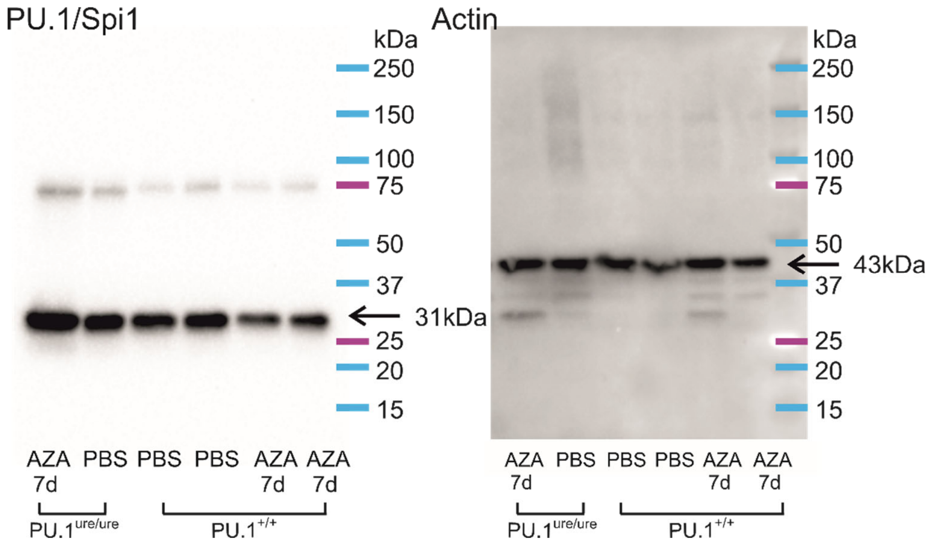

SFS2F legend: **AZA derepresses the *Pu.1* gene in PU.1$^{ure/ure}$ AML.** Protein levels of PU.1 and β-actin upon 1-week AZA treatment in 3 doses (150 µg/mouse), genotypes indicated. Western blot for PU.1 shows one dominant band at an expected mass of 31 kDa. We also observe one very faint band of 50-60 kDa, which is probably a non-specific band.

Supplementary Figure S3A

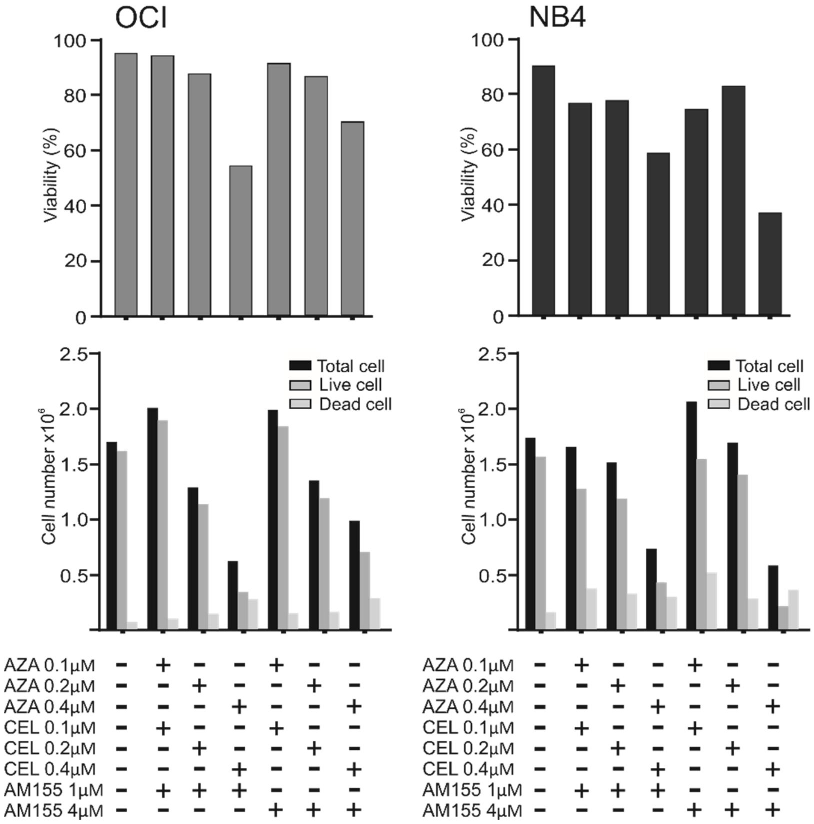

SFS3A legend: **Combined CEL/AZA/AM155 therapy in human AML.** Triplicate experiments. OCI-M2 shown on left, NB4 cells on right. 72 hr treatment with AZA, CEL, AM155 and their combinations, final concentrations indicated. Upper panel shows graphs of viability of cells. Bottom panel shows bar graphs representing Cell number of live and dead cells. Both were measured by Luna IITM Automated Cell Conter (Logos Biosystems).

Supplementary Figure S3B

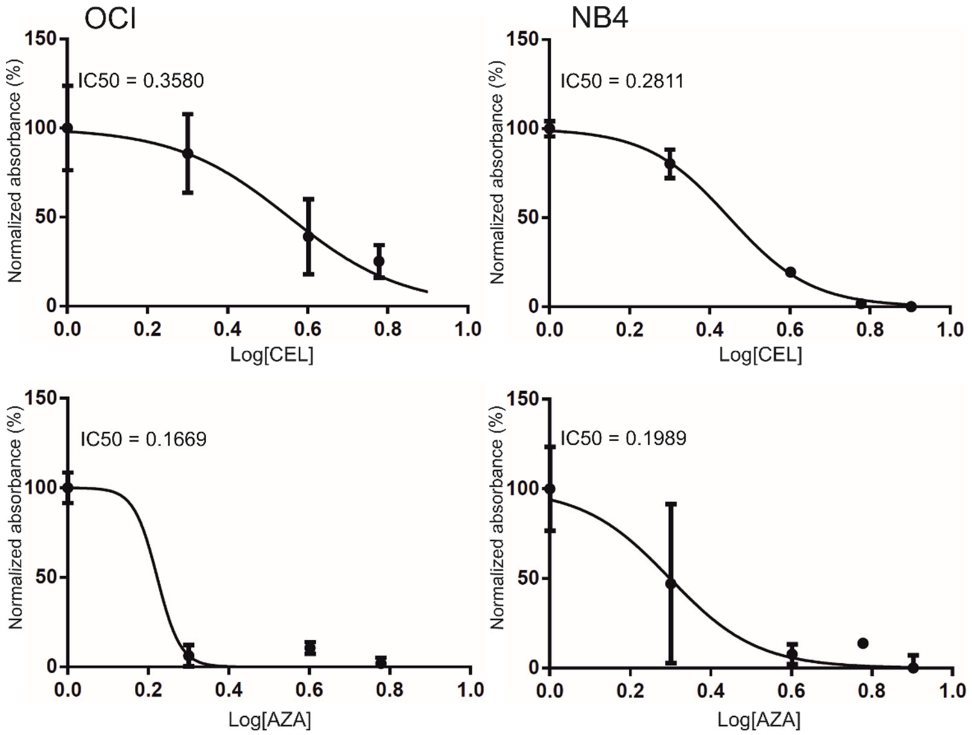

SFS3B legend: Inhibitory concentration (IC) with 50% effect (using the WST1 assay) in μM for CEL and AZA in two human AML cell lines (NB4, OCI-M2).

Supplementary Figure S3C

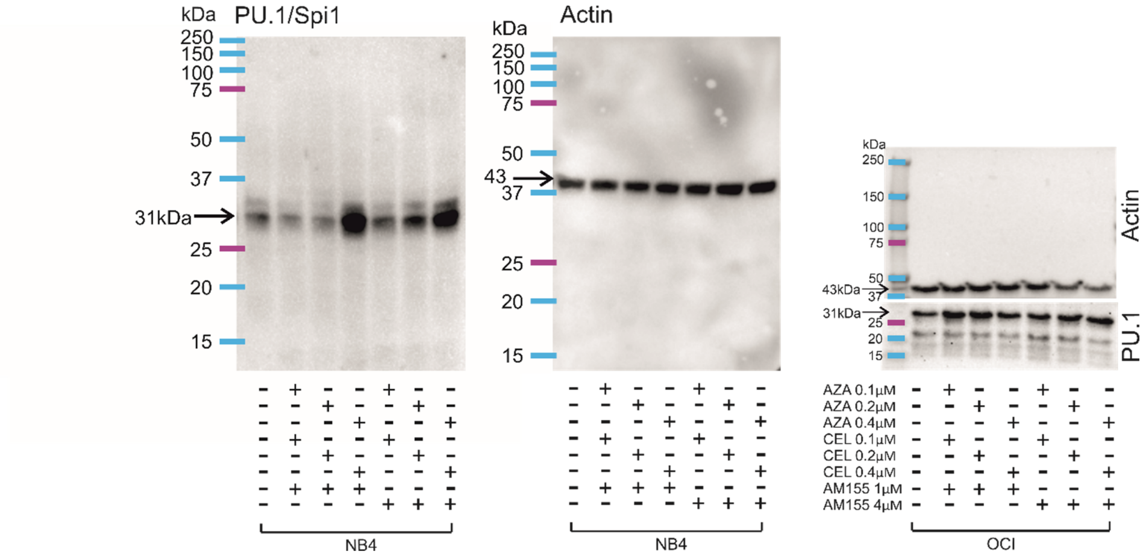

SFS3C legend: **Combined CEL/AZA/AM155 therapy in human AML.** Protein levels of PU.1 and β-actin upon 72 hr therapy with AZA, CEL, AM155; µM concentrations indicated.

Supplementary Figure S3D

D

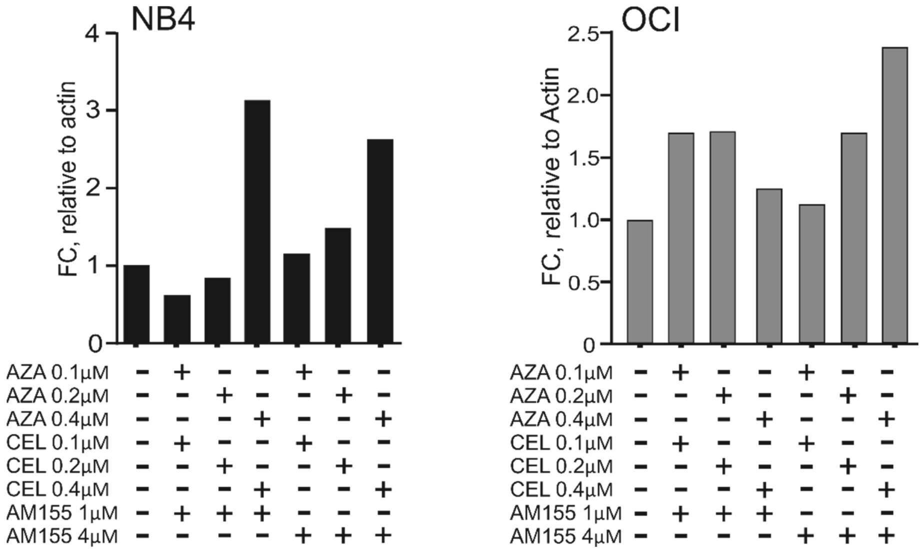

SFS3D legend**: Combined CEL/AZA/AM155 therapy in human AML.** Western blot (densitometry) for PU.1 protein and β-actin upon 72 hr therapy with AZA, CEL, AM155; μM concentrations indicated. Chemiluminescence was captured ba ChemiDocTM MP System equipped with Image LabTM software 5.2.1 (Bio-rad).

Supplementary Figure S3E

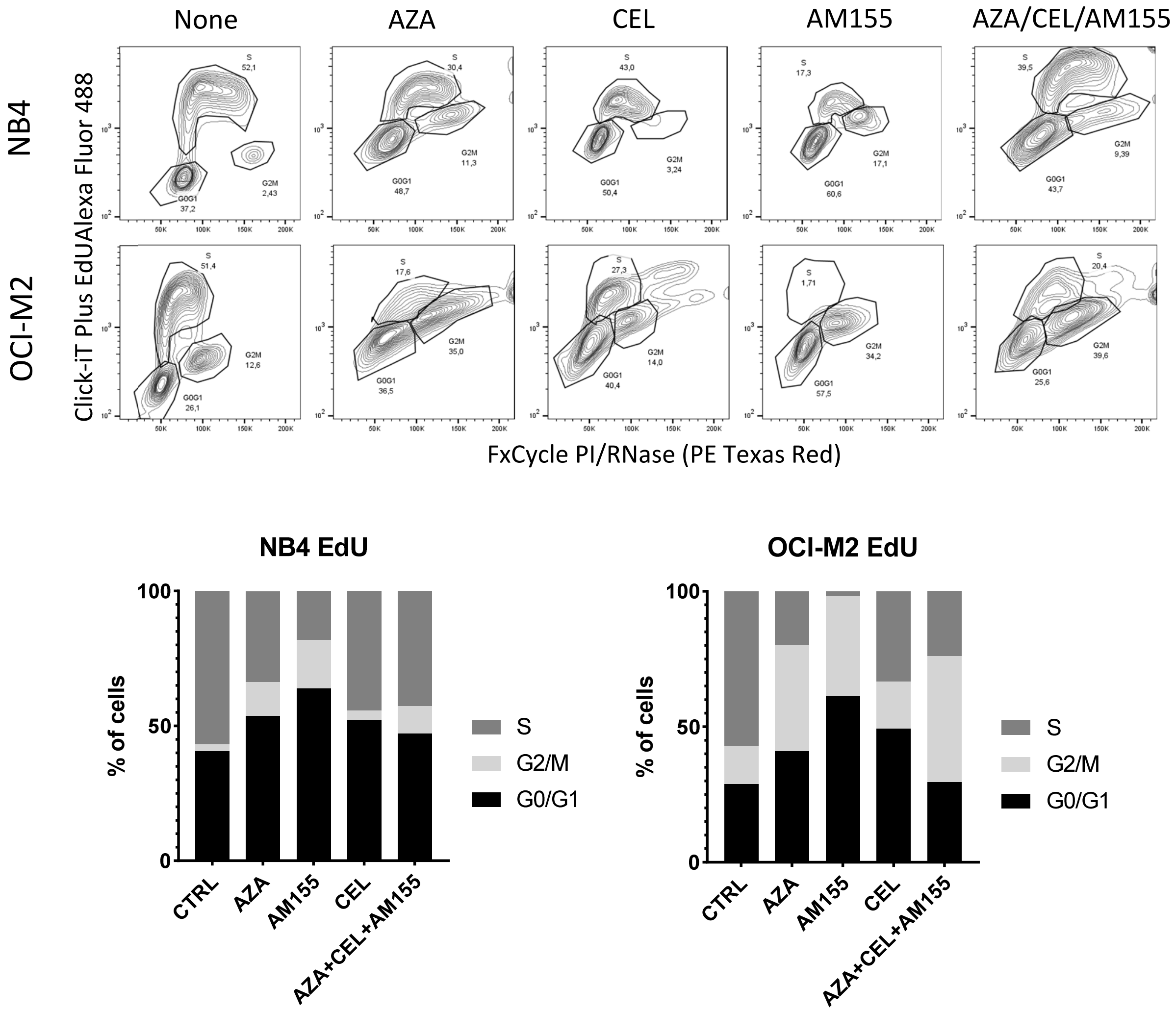

***SFS3E legend*: Combined CEL/AZA/AM155 therapy induce a cell cycle arrest at G0/G1 phase in human AML.** Dual parametr plot of Alexa Fluor® 488 Click-iT® Plus EdU Flow Cytometry Assay Kits, and FxCycle™ PI/Rnase upon 72 hr therapy with 0,4µM AZA, 0,4µM CEL, 4µM AM155 in two human AML cell lines (NB4 upper, OCI-M2 lower panel). Data were analyzed using an BD LSRFortessa™ Cell Analyzer, for detection of EdU with Alexa Fluor® 488 picolyl azide we used 488nm excitation with a green emission filter (530/30 nm). This figure combines DNA content with EdU; cells that are positive for both labels are in S-phase of the cell cycle. Bottom panel shows box graphs representing quantified values of the flow cytometry data.

Supplementary Figure S3F

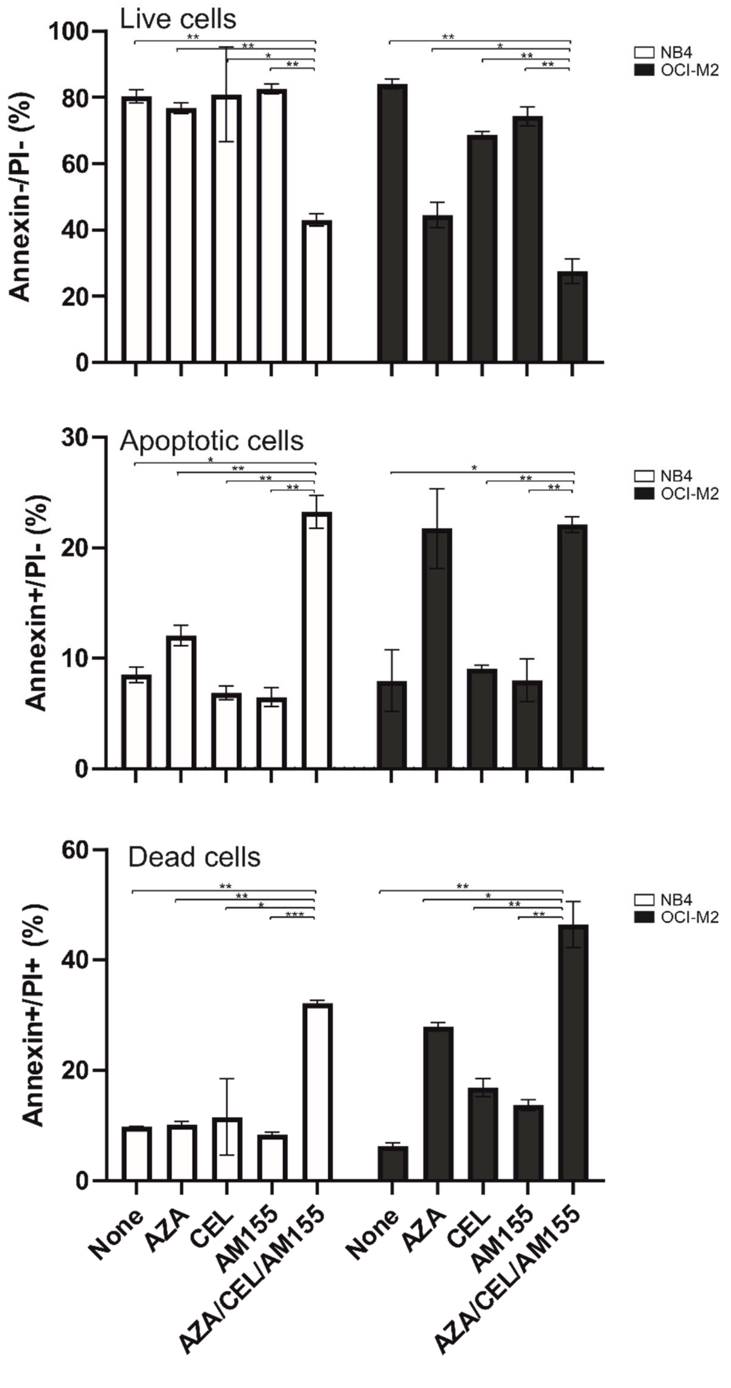

***SFS3F legend*: Combined CEL/AZA/AM155 therapy induce apoptosis in human AML.** NB4 and OCI-M2 AML cells stained with Annexin V/PI after 72 hr therapy with 0,4µM AZA, 0,4µM CEL, 4µM AM155. Data represent Mean ± SEM, P-values (t-test, unpaired, two-tailed). Data were analyzed using an BD LSRFortessa™ Cell Analyzer.

Supplementary Figure S4A

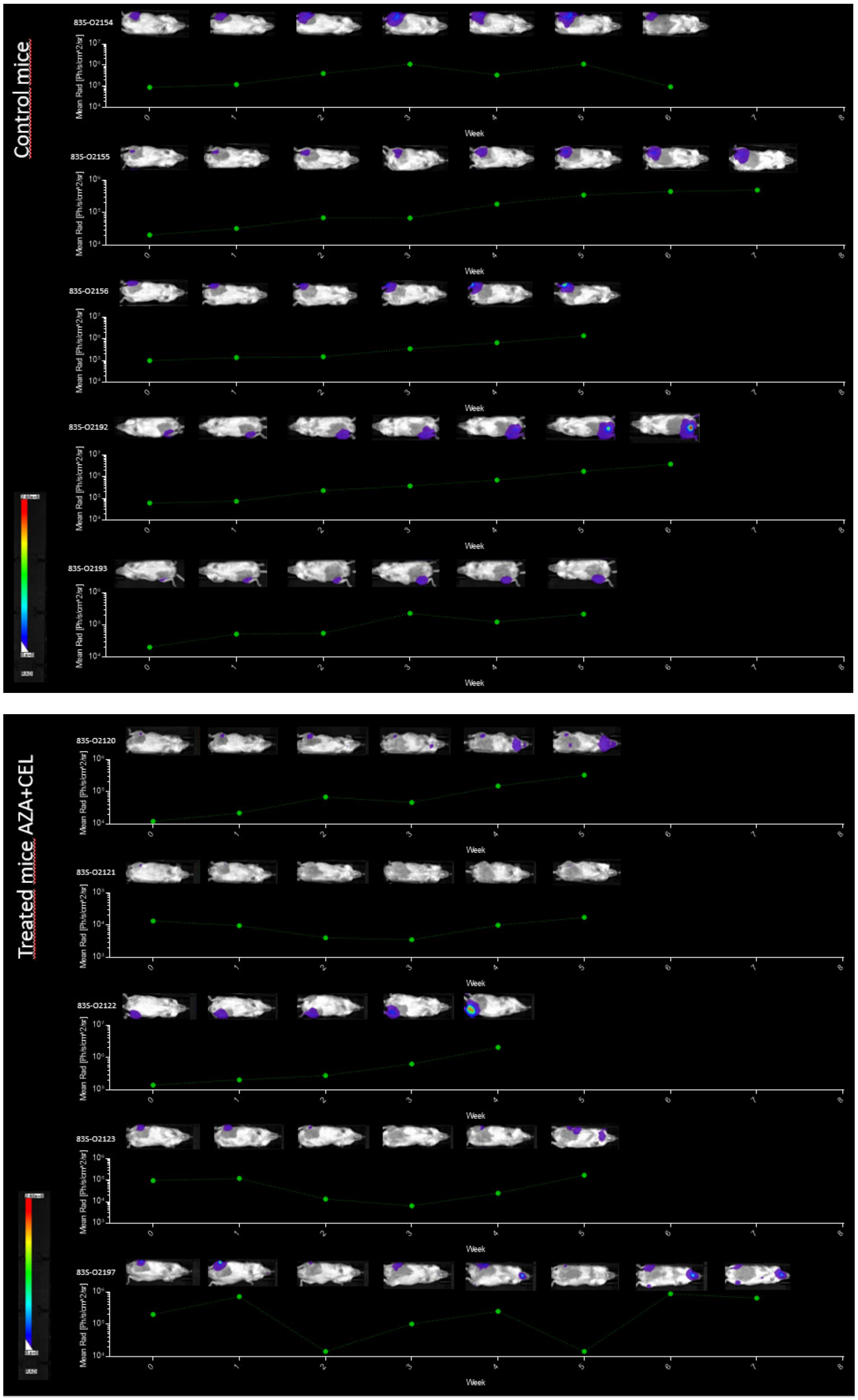

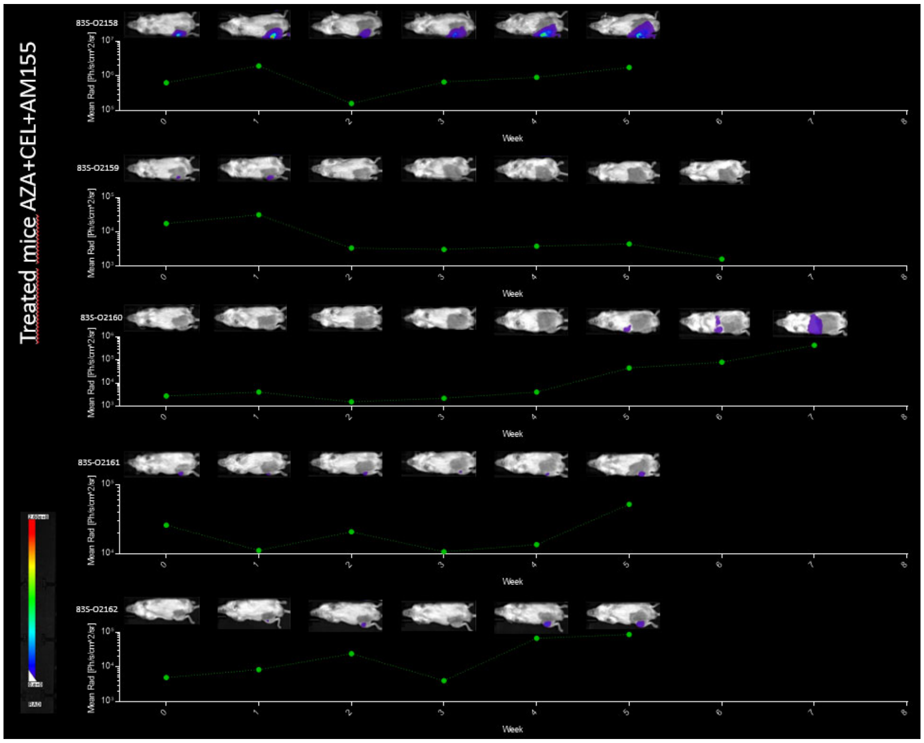

SFS4A legend: **Weekly bioimaging of mice with intraosseally administered OCI-M2 tumor cells:** either treated with vehicle or with AZA-CEL or 3-combination (AZA-CEL-AM155) at the given scheme. Luminiscence data summary is presented in the Fig 5C, for detail see M&M. We observed continous progression of tumor luminiscence intensity in the group of control mice (top panel) compared to treated mice marked by a a decline of tumor-associated luminiscence intensity either with combination of azacitidine (AZA) with celastrol (CEL) (middle panel) or in combination of AZA, CEL and AM155 (bottom panel).

Supplementary Figure S4B

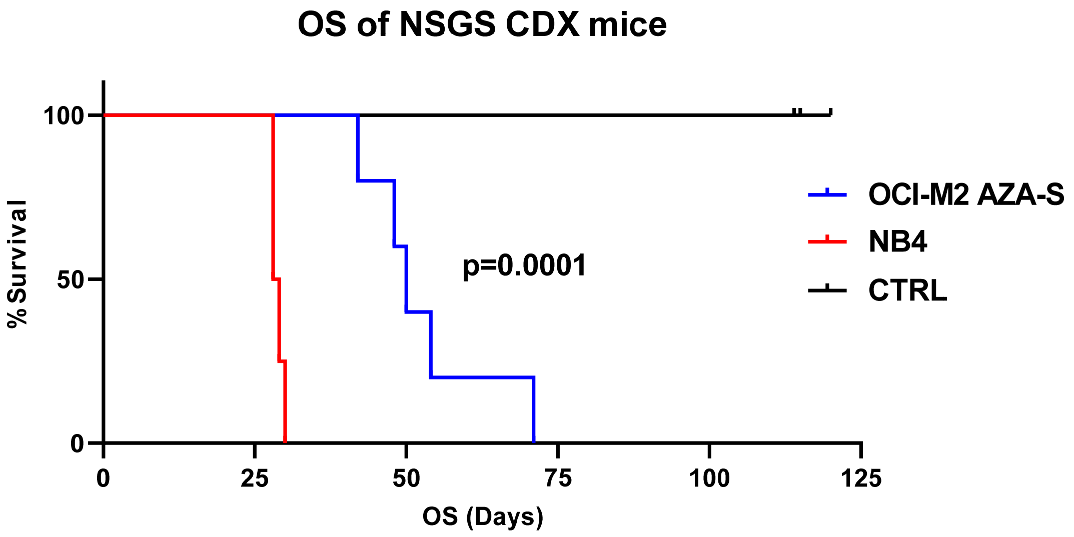

***SFS4B legend*: Survival curve of NSGS CDX mice with intraosseally administered tumor cells**: OCI-M2 (N=5) blue line, NB4 (N=5) red line and control mice (N=3) black line.
